# Supplementary material for: Analysis of Fast Fluorescence Kinetics of a Single Cyanobacterium Trapped in an Optical Microcavity
Source: Plants (Basel). 2023 Jan 30;12(3):607. doi: 10.3390/plants12030607 (PMC9919002; doi:10.3390/plants12030607)
Supplement: Supplementary file 1 [file plants-12-00607-s001.zip › plants-2054560-supplementary.pdf]

plants

Supplementary Information

Analysis of Fast Fluorescence Kinetics of a Single Cyanobacterium Trapped in an Optical Microcavity

T. Rammler[1,2], F. Wackenhut[1]*, J. Rapp[3], S. zur Oven-Krockhaus[1,2] and K. Forchhammer[3], K. Harter[2], A. J. Meixner[1]*

[1]Institute of Physical and Theoretical Chemistry, University of Tübingen, Tübingen, Germany
[2]Center for Plant Molecular Biology, University of Tübingen, Tübingen, Germany
[3]Interfaculty Institute of Microbiology and Infection Medicine, University of Tübingen, Tübingen, Germany
*Correspondence to: frank.wackenhut@uni-tuebingen.de, alfred.meixner@uni-tuebingen.de.

Microscopic imaging of *S. elongatus* and influence of DCMU on quantum yield

A confocal microscopy image of *S. elongatus* cells inside the microcavity is shown in Fig. S1A.

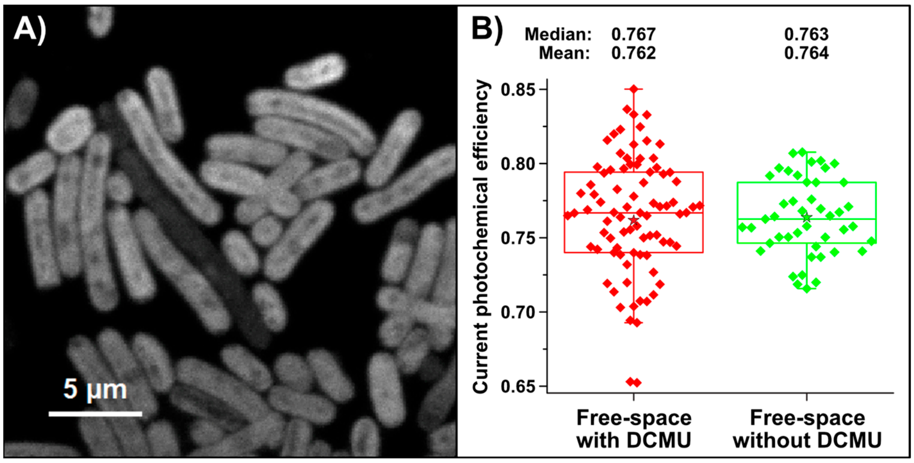

Figure S1. A) Confocal microscopy image of *S. elongatus* cells inside the microcavity. **(B)** Boxplot of the current photochemical efficiency of *S. elongatus* measured in free space, treated with DCMU (red) and untreated (green).

To ensure that DCMU did not significantly affect the measurements respective of the quantum yield, test measurements were performed with and without DCMU. Fig. S1B clearly shows that DCMU does not influence the measurements.

Absolute fluorescence values (F_0 and F_M)

Tabular values of the absolute, F_0-normalized and F_M-normalized quantum yield measurements in free space, resonant and off-resonant microcavity.

Table S1. Absolute, F_0-normalized and F_M-normalized F_0 and F_M values of quantum yield measurements in free space, resonant and off-resonant microcavity.

	Free space (counts)	Resonant (counts)	Off-resonant (counts)
F_0	11.73	13.69	9.37
F_M	49.27	49.03	37.48
Quantum Yield	0.762	0.721	0.749
F_0 (fixed, free space)	***11.73***	**11.73**	**11.73**

F_M (normalized)	49.27	42.22	46.92
F_0 (normalized)	11.73	13.76	12,32
F_M (fixed, free space)	***49.27***	**49.27**	**49.27**

Influence of a pulsed laser on *S. elongatus*

It became apparent during method optimization that pulsed lasers, working in the upper MHz range, were not suitable for our approach: The very short and intense laser pulses lead to disturbing light adaptation processes that resulted in a delayed onset of F_M. Light-adaptation responses were observed when a single cyanobacterium was exposed to a high intensity pulsed laser. Since the microcavity affected the quantum yield of single cells in the resonant state, we aimed to reveal whether the resonant state also affected the cyanobacteria light-adaptation processes caused by pulsed laser. Therefore, fluorescence time traces were acquired in a resonant microcavity and in free space as shown in Fig. S2A. The experiment showed a strikingly different fluorescence emission curve in the resonant state.

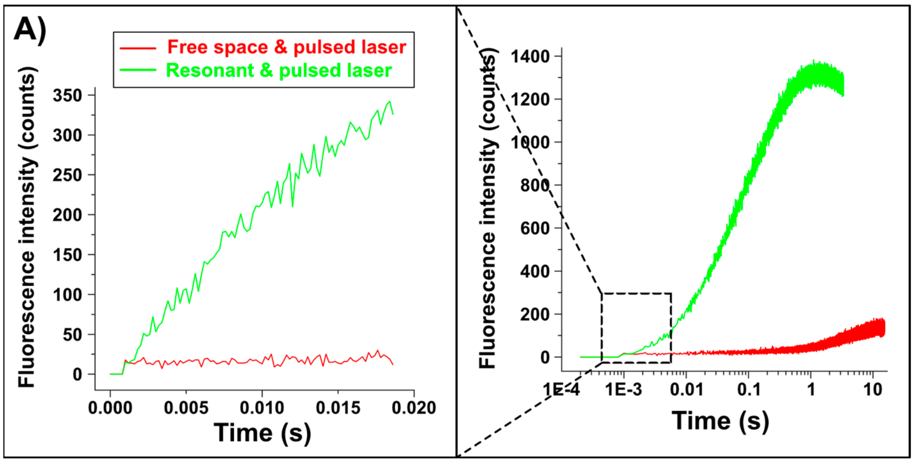

Figure S2. A) Two representative fluorescence curves of cyanobacteria in free space (red curve), in a resonant microcavity (green curve). Both measurements were carried out with a pulsed laser (repetition rate: 80 MHz, pulse duration: 80 ps) and an excitation intensity of 30 µW. The part on the left is zoomed.

The reason for such behavior must lie in the biology of the bacteria. Perhaps with so much laser power, the fluorescence increase is directly proportional to the F_M-level, as all P680s are immediately overloaded with photons. The true F_M value is presumed to be the same for both resonant and free space measurements. In free space, F_M is immediately reached in the millisecond range as expected and then remains constant for about one second. Subsequently, a further increase in fluorescence, beyond the F_M value is observed, which probably reflects light protection mechanisms induced by extremely high pulsed laser light. The complete overload of the PS2 with 30 µW per bacterium may have resulted in denaturation of the photosystem. The breaking of the chemical framework of the photosystem releases the individual chlorophyll molecules, which subsequently no longer act as complete photosystem, but like individual fluorophores. Thus, they behave by fluorescing more when they are excited more strongly, which is the normal, physical function of a free fluorophore.This is not the case with the intact photosystem.

In the microcavity in resonant state, the secondary fluorescence increased beyond F_M, was much stronger and started immediately upon pulsed laser onset. When the energy is trapped in the resonant state, the reflection at the mirrors and the reabsorption of the photons could have accelerated the photosystem denaturation process, leading to an immediate increase in fluorescence. The fact that the cells in free space can maintain F_M values

under this extreme photon pressure up to one second, illustrates a powerful yet unresolved capacity of short-term compensation of high excess energy.

The power of the experimental set-up is also illustrated by the detection of high-light induced photo-destruction caused by the resonant optical field in combination with high-power pulsed laser. In the absence of the resonant state, the time gaps between the pulses allow the cells to eliminate excess excitation energy, whereas, in the resonant state, the energy remains trapped within the system. Whether the steady increase in fluorescence indeed reflects denaturation of PS2 and which mechanism cells in free space dissipate this excess energy deserves further investigation.
